# Comparing Risk of New Onset Diabetes Mellitus in Chronic Kidney Disease Patients Receiving Peritoneal Dialysis and Hemodialysis Using Propensity Score Matching

**DOI:** 10.1371/journal.pone.0087891

**Published:** 2014-02-04

**Authors:** Che-Yi Chou, Chih-Chia Liang, Huey-Liang Kuo, Chiz-Tzung Chang, Jiung-Hsiun Liu, Hsin-Hung Lin, I.-Kuan Wang, Ya-Fei Yang, Chiu-Ching Huang

**Affiliations:** 1 Kidney Institute and Division of Nephrology, Department of Internal Medicine, China Medical University Hospital, Taichung, Taiwan; 2 Department of Medicine, China Medical University, Taichung, Taiwan; University of Florida, United States of America

## Abstract

Chronic kidney disease (CKD) patients are at risk for developing new-onset diabetes mellitus (NODM) even after hemodialysis (HD) and peritoneal dialysis (PD) treatment. It is not clear if the incidence for NODM is different in CKD patients receiving HD and PD. This study compared the risk of NODM in PD patients and HD patients.

**Methods:**

All HD and PD patients in Taiwan Renal Registry Database from 1997 to 2005 were included and all patients were followed to December 31, 2008. The risk of NODM was analyzed in PD patients and propensity score matched HD patients using logistic regression for early type NODM (< = 6 months after dialysis) and Cox regression for late type NODM (>6 months after dialysis).

**Results:**

A total of 2548 PD patients and 10192 HD patients who had no diabetes on the initiation of dialysis were analyzed. The incidence for NODM was 3.7 per 100 patient/year for HD and 2.4 for PD patients. HD patients are more at risk for developing early type NODM (p<0.001) with an adjusted odds ratio of 1.41 [95% confidence interval (CI) 1.12–1.78)]. HD patients are more at risk for late type NODM (p<0.001) with an adjusted hazard ratio of 2.01 (95% CI: 1.77–2.29). Patient’s age was negatively associated with risk of early type of NODM (p<0.001) but positively associated with risk of late type NODM (p<0.001).

**Conclusions:**

Chronic kidney disease patients receiving hemodialysis are more at risk for developing new-onset diabetes mellitus compared to those receiving peritoneal dialysis.

## Introduction

New onset diabetes mellitus (NODM) is a common complication in chronic kidney disease (CKD) patients receiving peritoneal dialysis (PD) or hemodialysis (HD) [1]. The development of NODM is linked to an increased overall mortality in CKD patients [2], [3]. The development of NODM in kidney transplant patients is associated with the use of immunosuppressant *i.e.* prednisolone and the risk factors of NODM in kidney transplant patients has been extensively studied [4]–[7]. Etiology of NODM in kidney transplant patients is different from that in HD and PD patients. Hyperglycemia is common in PD patients because of the use of glucose as the osmotic agents and is linked to a worse survival [2]. HD patients are also exposed to a glucose load because of the glucose in dialysate. However, very limited studies investigated the incidence, risk factors and outcomes of NODM in PD and HD patients. Understanding NODM risk factors may help to identify patients at risk for NODM, control patients’ blood glucose and may decrease NODM associated mortality. In addition, it is generally believed that the development of type 2 diabetes mellitus is linked to insulin resistance and β-cell dysfunction [8], [9]. The insulin resistance increased in the aging process [10], [11] and β-cell dysfunction can be caused by increased nutrient supply [12]. The incidence of NODM was 12.7% in 2 years in HD patients in US [1], 4% at 1 year and 21% in 9 years in Taiwan [13]. The risk for developing NODM [13] can be over-estimated since the competing events was not taken into consideration in the analysis [14]. Meanwhile, CKD 5 patients receiving PD are usually younger than those receiving HD [15]–[17] and HD patients may be more at risk for developing NODM. To compare the risk of NODM in CKD 5 patients receiving PD or HD, a propensity score matching for age and comorbidity was used. The risk for NODM was determined using competing-risks analysis in this study.

## Methods

This study was approved by the research and ethics committee of China Medical University Hospital. The data was obtained from Taiwan Society of Nephrology through institutional contact. All personal information was de identified before obtained. A total of 46596 chronic HD patients and 3516 PD patients (HD or PD for more than 1 month) in Taiwan Renal Registry Database from 1997 to 2005 were included and all patients were followed to December 31, 2008. The registry funded by the Department of Health, Taiwan, since 1987, collected information of all patients receiving dialysis from all dialysis units every year. It was a nationwide, non-government system, supervised by the Taiwan Society of Nephrology. Its data collection covers up to 95 percent of all dialysis patients in Taiwan [18]. This study was approved by the research and ethics committee of China Medical University Hospital. Patients receiving kidney transplant were excluded, as their risks for NODM are different from those receiving HD or PD. During the study period, 351 (1.2%) patients received kidney transplant, 788 (31%) PD patients changed to HD and 624 (2.4%) HD patients changed to PD. Most HD patients were treated using commercial available dialysate containing 100 or 200 mg/dl of glucose. A glucose free dialysate is rarely used in HD treatment because of an increased risk of hypoglycemia. The use of glucose sparing PD solution (icodextrin) in PD treatment was covered the Taiwan Health Insurance since 2006, very few (<1%) patients were treated using glucose sparing PD solution in the study period. Patients’ survival was recorded from the date of dialysis to the date NODM diagnosed, date of dialysis modes change (PD to HD, HD to PD, HD/PD to kidney transplant), death or December 31, 2008.

Underlying disease including chronic glomerulonephritis (CGN), hypertension, and others were diagnosed by a physician of nephrology. Comorbidity including hypertension (HTN), congestive heart failure (CHF), ischemic heart, cerebral vascular accident (CVA), liver disease, cancer, tuberculosis and others were reported by patients on the initiation of dialysis. Hypertension was defined as taking antihypertensives without regard to the actual measurement of blood pressure, or having a systolic blood pressure reading greater than 140 mm Hg or a diastolic blood pressure reading greater than 90 mm Hg [19]. Fasting blood glucose (FBG) was measured every 3 months and NODM was defined as at least two measurements of FBG ≥126 mg/dl and the date of the second measurement of FBG was considered as the date that NODM was diagnosed. The duration for developing NODM was recorded from date of dialysis to the date NODM diagnosed. Patients who developed NODM within 6 months after dialysis (HD or PD) were considered as early type NODM. Patients who developed NODM more than 6 months after dialysis were considered as late type NODM. Hematocrit, serum albumin, phosphate, calcium (corrected for serum albumin), and intact parathyroid hormone (i-PTH) were measured on the initiation of dialysis. Calcium phosphate product (CPP) was calculated as serum calcium multiple by serum phosphate.

### Statistical Analysis

Data are reported as mean ± SD or percent frequency, as appropriate. Testing for statistical significance was conducted using Student’s *t* test for parametric variables, chi-square test for categorical variables and Mann–Whitney U test for non-parametric variables. A propensity score was generated for each patient based on clinical factors that related to the selection of PD or HD. To increase the statistic power, the maximal number of HD patients matched is selected. The final data includes all non-diabetic PD patients and propensity score matched HD patients. Variables that are significantly different among patients who develop NODM were considered as risk factors for NODM. Risk factors of early type NODM were analyzed using multivariate logistic regression. Risk factors of late type NODM were analyzed using multivariate Cox proportional hazards regression. An adjusted odds ratio (OR) for early type NODM and adjusted hazard ratio (HR) for late type NODM was calculated. All statistical analysis was performed with Stata version 12 SE (Statacorp, Texas, USA). A p<0.05 was considered as significant.

## Results

### Propensity Score Matching

**Figure 1:**
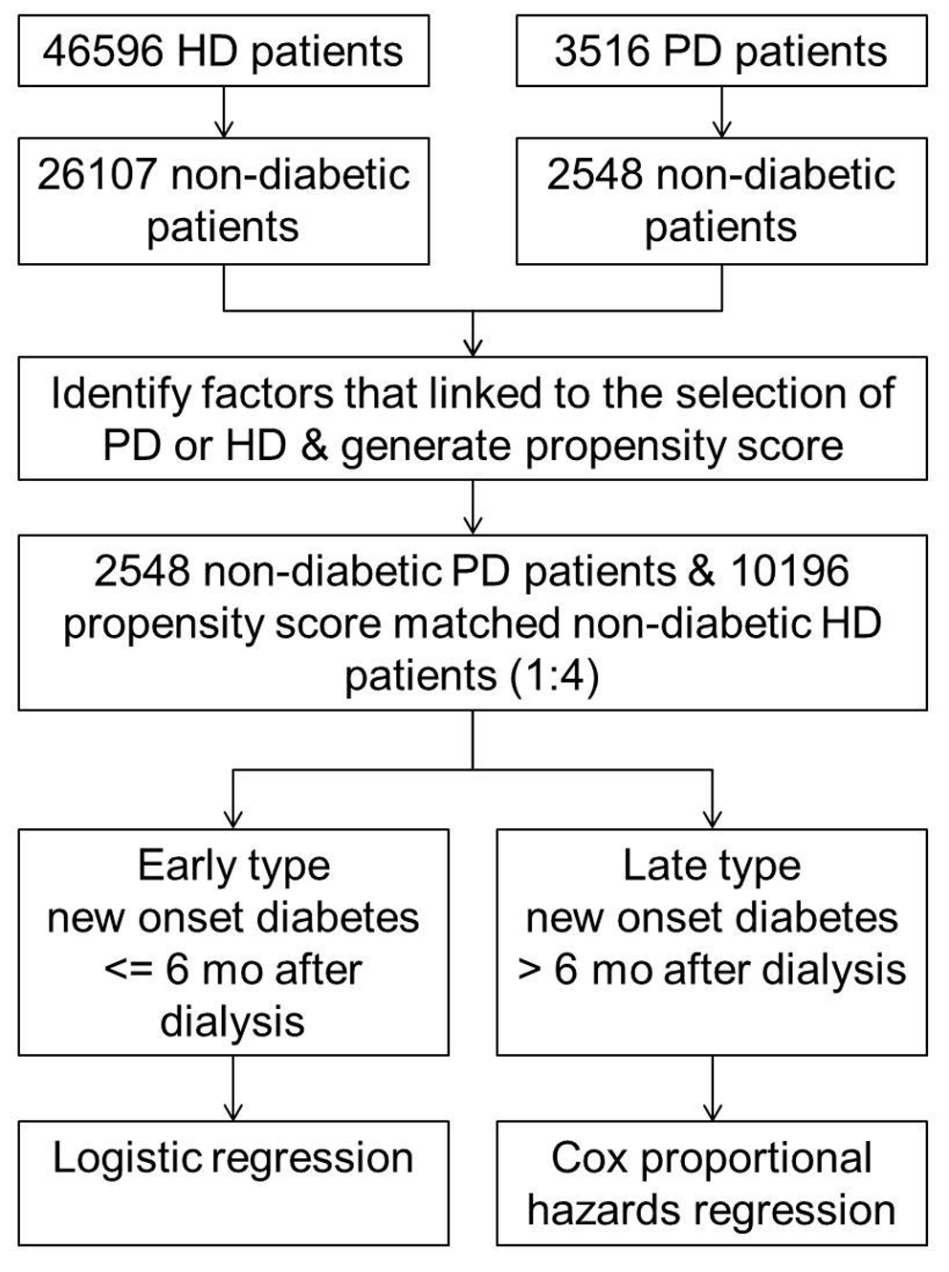
Scheme of study design. Factors used in the propensity score matching are patients’ age, gender, body weight, chronic glomerulonephritis (CGN) as an underlying disease, congestive heart failure (CHF), and number of comorbidity, hypertension (HTN), and patient’ hematocrit. A cohort consists of 2548 peritoneal dialysis and 10192 propensity score matched hemodialysis patients without diabetes was generated. The risk for new onset diabetes mellitus (NODM) was analyzed using logistic regression for early type NODM (patients who developed NODM < = 6 months after dialysis) and Cox proportional hazards regression for late type NODM (patients who developed NODM>month after dialysis).

Twenty-six thousand and one hundred seven of 46596 HD patients and 2548 of 3516 PD patients that had no diabetes on the initiation of dialysis were identified (Figure 1). PD patients were significantly younger than HD patients (51.9±14.8 years old *vs.* 59.6±3.5 years old, p<0.001) and thus a propensity score with matching for age was indicated. A propensity score based on patients’ age, gender, body weight, CGN as underlying disease, CHF, and number of comorbidity was generated as these variables were related to the selection of HD or PD. Patient’s hematocrit, HTN was associated with the development of NODM. Hematocrit and HTN was used in the propensity score matching. To increase the power of statistical analysis, a ratio of 1∶4 (PD: HD) was used, no matched cases were available in HD patients with a higher ratio. The analysis was performed in 2548 PD patients and 10192 propensity score matched HD patients (Table 1). The basal characteristics were not different between HD patients and PD patients. The incidence of NODM was 2.4 per 100 patients/year in PD patients and 3.7 per 100 patients/year in HD patients. The incidence of overall mortality was 5.5 per 100 patients/year in HD patients and 5.6 per 100 patients/year in PD patients.

**Table 1:** Clinical characteristics of 10192 hemodialysis (HD) and 2548 peritoneal dialysis (PD) patients.

	HD n = 10192		PD n = 2548		p
Age (year)	50.3	±14.5	50.2	±14.7	0.76
Follow-up (year)	5.9	±3.1	5.8	±2.7	0.14
NODM (per 100 pts/year)	3.7		2.4		–
Mortality (per 100 pts/year)	5.5		5.6		–
Male Gender	3692	(36.2)	916	(35.9)	0.80
Body weight (kg)	59.4	±4.5	59.3	±4.7	0.32
Underlying disease					
CGN	5913	(58)	1492	(58.6)	0.62
HTN	962	(9.4)	231	(9.1)	0.56
Others	3371	(33.1)	825	(32.4)	0.50
Co-morbidity (n, %)					
HTN	3779	(37.1)	948	(37.2)	0.91
CHF	471	(4.6)	116	(4.6)	0.88
Ischemic heart	428	(4.2)	94	(3.7)	0.25
CVA	185	(1.8)	43	(1.7)	0.66
Liver disease	289	(2.8)	75	(2.9)	0.77
Cancer	156	(1.5)	41	(1.6)	0.77
Tuberculosis	62	(0.6)	14	(0.5)	0.08
Other	717	(7.0)	175	(6.9)	0.77
Hematocrit (%)	28.8	±3.5	28.7	±3.7	0.20
Serum albumin (g/dl)	3.8	±0.4	3.8	±0.4	0.24
Calcium (mg/dl)	9.7	±0.7	9.7	±0.8	0.99
Phosphate (mg/dl)	5.1	±1.5	5.1	±1.2	0.99
CPP (mg/dl)^2^	49.5	±11.9	49.2	±12.9	0.26
FBG (mg/dl)	109	±57	108	±50	0.42
i-PTH (ng/dl)	277.8	±283.7	276.8	±262.5	0.87*

CGN: chronic glomerulonephritis, HTN: hypertension, CHF: congestive heart failure, CVA: cerebral vascular accident, FBG: fasting blood glucose, CPP: calcium-phosphate product, i-PTH: intact parathyroid hormone.

*Mann-Whitney U test.

### Risk Factor of NODM

Patients with NODM were older, had a shorter follow-up, and a higher mortality rate than patients without NODM (Table 2). Of 2568 NODM patients, 956 (37.2%) patients developed NODM within 6 months after dialysis and were considered as early type NODM. The prevalence of HTN as underlying disease (p<0.001) was higher but the comorbid HTN was lower in patients with NODM (p = 0.01). In biochemistry characteristics, patients who developed NODM had a lower hematocrit (p<0.001), serum albumin (p<0.001), phosphate (p<0.001), CPP (p<0.001), i-PTH (p<0.001, Mann-Whitney U test), but a higher FBG (p<0.001). Risk factors of early type NODM were analyzed using univariate logistic regression (data not shown) and factors with a p<0.05 were further analyzed using multivariate logistic regression (Table 3). HD (*v.s.* PD) was associated with an increased risk of early type NODM (p = 0.004) with an OR of 1.41(95% CI: 1.12–1.78). Patient’s age (p<0.001) and male patients (p<0.026) was independently associated with a decreased risk of early type NODM. Patient’s hematocrit (p = 0.016), serum albumin (p<0.001), and iPTH (p = 0.013) was positively linked to an increased risk of early type NODM. The OR was 0.885 (95% CI: 0.829–0.945) for every 10 years older, 0.821 (95% CI: 0.691–0.976) for male gender, 1.03 (95% CI 1.01–1.05) for every 1% hematocrit increment, 1.37 (95% CI: 1.14–1.65) for every 1 gm/dl increase of serum albumin, and 1.05 (95% CI: 1.01–1.09) for every 100 ng/dl increase of iPTH.

**Table 2:** Clinical characteristics of patients with and without new onset diabetes mellitus (NODM).

	NODM(−) n = 10172		NODM(+) n = 2568		p
Age (year)	48.3	±14.1	56.6	±13.7	<0.001
Follow-up (year)	6.2	±2.8	4.8	±2.7	<0.001
Male gender n(%)	3650	(35.9)	958	(37.3)	0.45
HD n(%)	7975	(78.4)	2217	(86.3)	<0.001
Mortality n(%)	2841	(27.9)	1281	(49.9)	<0.001
Weight (kg)	69.8	±8.5	70.1	±7.7	0.10
Underlying disease n(%)					
CGN	5915	(58.1)	1490	(58)	0.91
Hypertension	902	(8.9)	291	(11.3)	<0.001
Others	3383	(33.3)	813	(31.7)	0.12
Co-morbidity n(%)					
Hypertension	3829	(37.6)	898	(35)	0.01
CHF	455	(4.5)	132	(5.1)	0.15
Ischemic heart	428	(4.2)	94	(3.7)	0.21
CVA	179	(1.8)	49	(1.9)	0.61
Liver disease	283	(2.8)	81	(3.2)	0.31
Cancer	155	(1.5)	42	(1.6)	0.68
Tuberculosis	57	(0.6)	19	(0.7)	0.29
Others	718	(7.1)	174	(6.8)	0.62
Hematocrit (%)	29.4	±3.6	28.8	±3.7	<0.001
Albumin (g/dl)	3.9	±0.4	3.8	±0.5	<0.001
Phosphate (mg/dl)	5.1	±1.3	4.9	±1.5	<0.001
Calcium (mg/dl)	9.6	±0.8	9.6	±0.8	0.14
CPP (mg/dl)^2^	48.9	±13.2	47.1	±14.5	<0.001
FBG (mg/dl)	98	±34	169	±68	<0.001
i-PTH (ng/dl)	272.6	±257.6	243.7	±262.3	<0.001*

HD: hemodialysis, CGN: chronic glomerulonephritis, HTN: hypertension, CHF: congestive heart failure, CVA: cerebral vascular accident, FBG: fasting blood glucose, CPP: calcium-phosphate product, i-PTH: intact parathyroid hormone.

*Mann-Whitney U test.

**Table 3:** Odds ratio of risk factor for early type new-onset diabetes among peritoneal dialysis (PD) or hemodialysis (HD) patients using multivariate logistic regression.

	OR	95% C.I		p
HD (v.s. PD)	1.41	1.12	1.78	0.004
Age (every 10 years older)	0.885	0.829	0.945	<0.001
Male gender	0.821	0.691	0.976	0.026
HTN	0.899	0.751	1.08	0.249
Hematocrit	1.03	1.01	1.05	0.016
Serum albumin	1.37	1.14	1.65	0.001
CPP	0.999	0.993	1.005	0.674
iPTH (every 100 ng/dl increment)	1.05	1.01	1.09	0.013

CPP: calcium-phosphate product, HTN: hypertension, CPP: calcium phosphate product.

**Figure 2:**
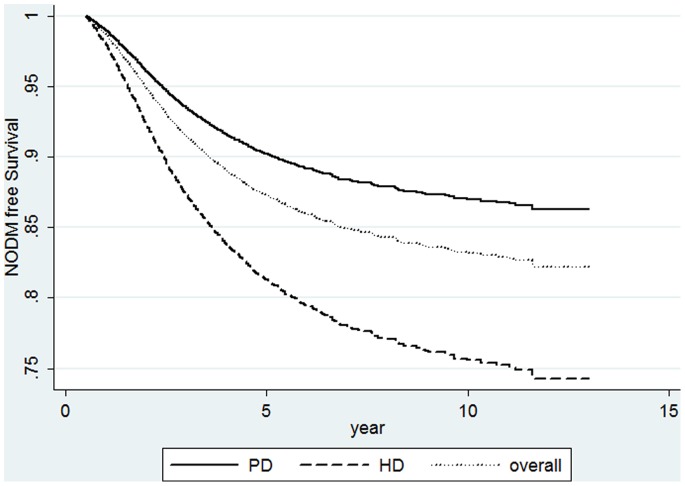
A new-onset diabetes free survival curves of hemodialysis (HD), peritoneal dialysis (PD), and all patients with adjustments for patients’ age, gender, hypertension, hematocrit and serum albumin.

Risk factors for late type NODM were analyzed using univariate Cox proportional hazards regression (data not shown) and factors with a p<0.05 were further analyzed using multivariate Cox proportional hazards regression (Table 4). HD was associated with an increased risk for late type NODM (HR: 2.01 95% CI: 1.77–2.29, p<0.001, Figure 2). Patient’s age (HR: 1.46 for every 10 years older, 95% CI: 1.41–1.51, p<0.001) and male patients (HR 1.27, 95% CI: 1.16–1.39, p<0.001) was independently linked to an increased risk for late type NODM. An increased hematocrit (p<0.001) and serum albumin (p<0.001) was independently linked to a decreased risk for late type NODM.

**Table 4:** Hazard ratio (HR) of risk factor for late type new-onset diabetes in chronic kidney disease patients receiving peritoneal dialysis (PD) or hemodialysis (HD) in multivariate Cox proportional hazards regression.

	HR	95% C.I		p
HD (v.s. PD)	2.01	1.77	2.29	<0.001
Age (every 10 years older)	1.46	1.41	1.51	<0.001
Male gender	1.27	1.16	1.39	<0.001
HTN	0.855	0.782	0.934	0.001
Hematocrit	0.973	0.960	0.986	<0.001
Serum albumin	0.565	0.505	0.633	<0.001
CPP	1.00	0.996	1.003	0.872
iPTH	0.995	0.977	1.013	0.571

FBG: fasting blood glucose, CPP: calcium-phosphate product.

### NODM and Overall Mortality

The development of NODM was associated with an increased mortality risk (Table 5) with a HR of 1.42 (95% CI 1.32–1.52, p<0.001). Male patients (p<0.001), older patients (p<0.001) and patients with more comorbidity (p<0.001) had increased overall mortality. Patients with CGN as the cause of CKD 5 (p<0.001) and patients with HTN (p<0.001) had a decreased mortality risk. An elevated serum albumin (p<0.001), CPP (p = 0.005) and hematocrit (p<0.001) were independently associated with a decreased overall mortality.

**Table 5:** Hazard ratio of prognostic factors in chronic kidney disease 5 patients receiving hemodialysis or peritoneal dialysis.

	HR	95.0% CI		p
NODM	1.42	1.32	1.52	<0.001
Age (every 10 years older)	1.60	1.56	1.64	<0.001
Male gender	1.54	1.44	1.64	<0.001
CGN	0.850	0.797	0.906	<0.001
HTN	0.701	0.640	0.768	<0.001
Number of comorbidity	1.25	1.19	1.32	<0.001
Serum albumin	0.407	0.377	0.441	<0.001
CPP	0.996	0.994	0.999	0.005
Hematocrit	0.950	0.940	0.959	<0.001

NODM: new onset diabetes mellitus, HTN: hypertension, CGN: chronic glomerulonephritis, CPP: calcium-phosphate product.

## Discussion

In this observational cohort study, the incidence of NODM of chronic kidney disease 5 patients receiving PD was 2.4 per 100 patients/year and 3.7 per 100 patients/year in those receiving HD. Compared to PD patients, HD patients had a 41% increased risk for developing of NODM in 6 months after HD and 2-fold increased risk for developing NODM more than 6 months after HD. The association between HD and risk of NODM was independent of patient’s age, gender, comorbid hypertension, hematocrit, and serum albumin. The elevated NODM risk in HD patients may be explained by HD treatment *per se*. The blood-membrane interaction in HD treatment can induce increased cytokines [20] such as C-reactive protein [21] and interleukins-6 [22] in HD patients but not in patients treated with PD. Chronic inflammation indicated by an elevated C-reactive protein or interleukin-6 plays a critical role in the development of diabetes [23]–[26]. The increased risk for NODM in HD patients may be explained by the chronic subclinical inflammation induced by the HD dialyzer. The decreased risk for NODM among PD patients may be related to their physical activity. Most of the PD patients take more responsibility in their treatment as they need to performed PD exchanges on their own. Thus, PD patients may have better physical activities in daily life than HD patients. Increased physical activities may prevent the development of diabetes [27].

It is of note, that patient’s age was negatively associated with early type NODM and positively associated with late type NODM. The increased risk for late type NODM with age can be explained by an increased insulin resistance in aging process [10]. Patients who developed early type NODM were older and had a higher mortality rate. Patient’s overall mortality was highest in the first 3 to 6 months of dialysis [16]. Therefore, patients without early type NODM may have a better survival. This may explain the association between patient’s age and risk of early type NODM.

Although glucose load leads to an elevated FBG glucose loading is not a risk factor of NODM [9]–[11]. Nutrition supplements had been considered as a risk factor of NODM [12]. An increased serum albumin and hematocrit was linked to an increase risk of early type NODM (Table 3). This finding may indirectly support the influence of nutrition supplements on the development of NODM. In addition, chronic inflammation may play an important role in the development of late type NODM. This is also supported by the negative association between hematocrit, albumin and risk of NODM (Table 4). Glucose is one of the components in dialysate used in HD [28], [29] and PD, the FBG measurement may not be “truly” fasting blood glucose. Thus a FBG > = 200 mg/dl to defined diabetes was performed. The incidence of NODM decreased, but patients receiving HD was consistently associated with an increased risk of NODM than those receiving PD. Based on the patient’s age of diabetes diagnosed, most of NODM patients may have type 2 diabetes. As oral-hypoglycemic agents was not available in the registry data, it is unknown if insulin is needed in these NODM patients. In addition, the incidence of NODM reported in this study is lower than the incidence reported in the previous study [1], [13]. The low incidence of NODM in the patients receiving HD/PD may be explained by the ethnic and genetic differences [30]–[33]. Patients with NODM had a 38% increased risk of death that is similar to the risk reported in Taiwan National Health Insurance data [13]. The association between NODM and patients’ mortality is independent of age, underlying disease, HTN, albumin, CPP and hematocrit (Table 4). This finding is also supported by previous studies [1], [3], [16].

A propensity score matching was critical in the investigation of NODM risk in CKD 5 patients because younger CKD 5 patients are more likely to be treated with PD. The propensity score matched HD patients had a similar age, body weight, ratio of underlying disease and comorbidity to PD patients (Table 1 & Figure 1). As the risk of NODM was significantly higher in propensity score matched HD patients, patient selection (age, underlying disease and comorbidity) bias has a minimal effect on our finding.

Obesity, especially an increased visceral fat distribution [34]–[37], is linked to insulin resistance and the development of diabetes [34], [35], [38]. Body mass index (BMI) is one of the most commonly used anthropometric measurements of obesity; however, BMI was not calculated, as patients’ height is not available in our data. Waist to hip ratio or waist to height can be good indicators for central obesity [39], but waist and hip circumferences are not available. These are potential limitation of our study. Patients’ body weight was taken into consideration in propensity score, but did not significantly contribute to the development of NODM in patients treated with HD or PD. In addition, anti-hypertensives such as beta-blocker is linked to an increased risk of NODM [40], but anti-hypertensive treatment was not recorded in the data. The role of anti-hypertensives in the development of NODM in patients on receiving PD and HD remains unknown.

In conclusion, the risk for developing new onset diabetes mellitus is 2.4 per 100 patients/year in CKD 5 patients receiving peritoneal dialysis and 3.7 per 100 patients/year in those receiving hemodialysis. HD patients are more at risk for developing new onset diabetes than PD patients. Patient’s age, serum albumin, and hematocrit is independently linked to the development of NODM. The development of NODM is associated with an increased overall mortality in chronic kidney disease patients.
